# Evaluation of the Biolabo Turbidimetric Assay for Automated Determination of Haemoglobin A1c

**DOI:** 10.3390/diagnostics15080969

**Published:** 2025-04-10

**Authors:** Lorenza Fagnani, Simonetta De Angelis, Pierangelo Bellio, Patrizia Frascaria, Rita Tennina, Giovanni Alloggia, Francesco Gentile, Alessandra Piccirilli, Mariagrazia Perilli, Giuseppe Celenza

**Affiliations:** 1 Department of Biotechnological and Applied Clinical Sciences, University of L’Aquila, 67100 L’Aquila, Italy; lorenza.fagnani@univaq.it (L.F.); giovanni.alloggia@graduate.univaq.it (G.A.); alessandra.piccirilli@univaq.it (A.P.); mariagrazia.perilli@univaq.it (M.P.); giuseppe.celenza@univaq.it (G.C.); 2 Clinical Laboratory, Regional Hospital “San Salvatore”, 67100 L’Aquila, Italy; pfrascaria@asl1abruzzo.it (P.F.); rtennina@asl1abruzzo.it (R.T.); francescogentile986@gmail.com (F.G.)

**Keywords:** analytical performance, biomarker, diabetes monitoring, HbA1c measurement, HPLC alternative, immunoturbidimetric assay, laboratory analysis

## Abstract

**Background/Objectives**: The determination of glycated haemoglobin (HbA1c) is a cornerstone of the diagnosis and management of diabetes mellitus, serving as a reliable biomarker for assessing long-term glycaemic control. While high-performance liquid chromatography (HPLC) is regarded as the gold standard for HbA1c measurement, its widespread adoption is limited by high costs, operational complexity, and resource requirements. Alternative methodologies, including immunoturbidimetric assays, have garnered interest as practical solutions. This study evaluates the analytical performance of an immunoturbidimetric method for HbA1c determination and its comparability with a validated HPLC method. **Methods**: The evaluation process was conducted in accordance with the Clinical and Laboratory Standards Institute (CLSI) guidelines. The results from 178 human sample leftovers, covering the medical decision range, were compared with those obtained using the HPLC-based Menarini ADAMS A1c HA-8180T system. The analytical performance regarding repeatability and within-laboratory imprecision was also assessed. The probability risk of misinterpreting the analytical results was also calculated. **Results**: The Passing–Bablok regression indicated a strong correlation between the two methods, with a slope of 1.00 (95% CI: 1.00 to 1.04). The Bland–Altman analysis confirmed minimal systematic differences, showing a mean bias of −0.07% for NGSP and −0.74 mmol/mol for IFCC, both falling within the predefined total allowable error (ATE) limits. Imprecision studies demonstrated excellent repeatability and intermediate precision, with coefficients of variation (*CV*) ranging from 0.68% to 2.4% across all levels. The risk assessment of diagnostic misinterpretation indicated minimal deviation from an ideal analytical system, in which the measurement uncertainty was regarded as zero. **Conclusions**: The findings establish the immunoturbidimetric method as a reliable and cost-effective alternative to HPLC for routine HbA1c determination. Its strong analytical performance, combined with operational efficiency, makes it a valuable tool for laboratories, particularly in resource-limited settings, enhancing access to high-quality diabetes monitoring.

## 1. Introduction

Diabetes mellitus is a growing global health challenge and one of the leading causes of morbidity and mortality worldwide. Its prevalence continues to rise in both developing and developed countries [1], and the World Health Organization (WHO) foresees a 170% increase in incidence in developing countries [1,2]. This increase emphasises the urgent need for effective diagnostic and monitoring strategies. Accurate and timely diagnostic tools are vital for preventing complications, yet up to 50% of individuals with diabetes remain undiagnosed, particularly in rural and hard-to-reach settings [3]. Haemoglobin A1c (HbA1c), also known as glycated haemoglobin, stands out as the most accurate and widely used biomarker for the monitoring and management of diabetes because of its ability to reflect long-term glycaemic control over the past two to three months [1,4,5]. Unlike daily glucose measurements that capture short-term fluctuations, HbA1c provides a stable and integrated measure of blood glucose levels, as it forms through a non-enzymatic glycation process [6]. During this process, glucose molecules in the bloodstream bind covalently to the N-terminal valine of the haemoglobin β-chain within erythrocytes, creating a glycohaemoglobin complex. Since the lifespan of red blood cells is approximately 120 days, HbA1c levels correlate closely with the average blood glucose concentrations over this period, providing a more reliable reflection of the metabolic status of a patient [6,7]. However, it is important to note that HbA1c has reduced diagnostic accuracy in clinical conditions affecting erythrocyte lifespan, such as iron deficiency anaemia or haemolytic anaemia, regardless of the analytical method used, representing a significant limitation in certain patient populations [8]. The clinical significance of HbA1c lies in its robust association with microvascular and macrovascular complications, including nephropathy, retinopathy, neuropathy, and cardiovascular disease [1,9,10], often leading to severe consequences if glycaemic control is not managed effectively [4,11]. Landmark studies evidenced by the Diabetes Control and Complications Trial (DCCT) and the UK Prospective Diabetes Study (UKPDS) have demonstrated the clinical significance of maintaining HbA1c levels below target thresholds to reduce the risk of diabetes-related complications [12,13]. A 1% reduction in HbA1c reduces the risk of microvascular complications by approximately 21% and macrovascular complications by 14%, underscoring the importance of stringent glycaemic control [13,14]. As a result, major health organisations, including the WHO and the American Diabetes Association (ADA), recommend HbA1c testing for both the diagnosis and monitoring of diabetes mellitus [1], with targets generally set below 7% for most patients to minimise complications and with even lower targets (e.g., below 6.5%) suggested for certain populations who may benefit from stringent control without the risk of hypoglycaemia [4,12,15,16,17,18]. In addition, higher baseline levels of HbA1c are correlated with an increased future risk of diabetes, even in individuals without an initial diagnosis [19]. Thus, HbA1c measurement has expanded beyond a monitoring tool, establishing itself as an essential component of diabetes screening and prevention strategies [5]. Consequently, accurate and consistent HbA1c measurement techniques are necessary, as test results directly inform the therapeutic adjustments required to meet individual glycaemic targets. The widespread clinical relevance of HbA1c in diabetes management has led to the development of several analytical methods [1,12,13,20], including cation-exchange chromatography, affinity chromatography, enzymatic assays, and immunoassays [21,22]. Initially, the lack of harmonisation among these methods resulted in significant variability in outcomes, creating confusion within both clinical and research communities. To address this issue, an international agreement has defined the HbA1c measurand as “all haemoglobin molecules having a special hexapeptide in common, which is the stable adduct of glucose to the amino-terminal valine of the haemoglobin β-chain (βN-1-deoxyfructosyl-hemoglobin)” [23]. Starting from the measurand definition, standardisation efforts led by the National Glycohemoglobin Standardization Program (NGSP) and the International Federation of Clinical Chemistry and Laboratory Medicine (IFCC) have been instrumental in establishing consistent reference values and calibration methods, which facilitate the comparability of results across laboratories and testing platforms [24,25,26,27]. There are two principal methods for reporting HbA1c, as follows: the National Glycohemoglobin Standardisation Program (NGSP) method, which expresses values as percentages (%), and the International Federation of Clinical Chemistry (IFCC) method, which reports values in mmol/mol to standardise international measurements [28] and ensure traceability and accuracy, enabling the comparability of results across assay types [29]. Differences between NGSP and IFCC values are resolved through a conversion formula, ensuring clinical relevance [28]. HbA1c measurements can exhibit minor variability due to methodological differences, with most methods reporting variations of 0.2–0.3%, although more significant discrepancies have been observed [30,31]. However, this standardisation of HbA1c results across all available techniques is essential for ensuring consistency and comparability in clinical practice worldwide [1,22]. High-performance liquid chromatography (HPLC) is widely regarded as the gold standard in HbA1c quantification because of its accuracy and specificity, including the ability to separate haemoglobin fractions and minimal interference from common haemoglobin variants [1,11]. However, its widespread adoption is limited by high costs and time consumption, as well as the need for trained technologists and expensive apparatuses, making it non-affordable for routine use in all laboratories [12,32,33]. To address these limitations, the scientific community has continued to refine alternative methods, with a particular focus on immunoassays, offering more practical solutions for HbA1c analysis, combining efficiency with cost-effectiveness, and maintaining accuracy. Emerging technologies, such as immunoturbidimetric assays [1,20,34], have gained traction as cost-effective and efficient alternatives for HbA1c measurement. These methods, calibrated to IFCC standards, provide rapid, reliable results that are suitable for diverse healthcare settings.

This study aims to evaluate the analytical performance of an immunoturbidimetric method applied to the Biolabo Kenza 450TX clinical chemistry analyser as a potential practical alternative to HPLC for measuring HbA1c.

## 2. Materials and Methods

### 2.1. Guidelines for the Evaluation Process

The evaluation process was performed in accordance with the Clinical and Laboratory Standards Institute’s guidelines. Specifically, CLSI EP05-A3 “Evaluation of Precision of Quantitative Measurement Procedures, 3rd Edition” [35], CLSI EP09C “Measurement Procedure Comparison and Bias Estimation Using Patient Samples, 3rd Edition” [36], and CLSI EP21 “Evaluation of Total Analytical Error for Quantitative Medical Laboratory Measurement Procedures, 2nd Edition” [37].

### 2.2. Analysers Description

The Kenza 450TX (K450TX), developed by Biolabo SAS (Maizy, France), is a fully automated, random-access biochemistry analyser designed for high-throughput laboratory settings. Capable of performing up to 420 tests per hour, the system supports a broad range of routine biochemistry applications. In addition, an optional ion-selective electrode (ISE) module is available for the measurement of sodium (Na), potassium (K), chloride (Cl), and lithium (Li), expanding its diagnostic versatility. The K450TX features 120 independent reagent positions, organised into two permanently cooled trays, each accommodating up to 60 positions. These trays are compatible with a variety of vial sizes (small, medium, and large) and microvolume adapters, providing flexibility for diverse testing requirements. To ensure operational safety and efficiency, the system automatically detects and resolves reagent incompatibilities.

For sample management, the analyser offers 98 positions dedicated to samples, controls, and calibrators, facilitating the simultaneous processing of diverse diagnostic needs. An integrated “STAT” function prioritises urgent samples, streamlining workflow in critical situations.

The system employs 50 long-life reading cuvettes, which are continuously cleaned by a high-performance washing station equipped with five needles (three double and two single) using water and specialised cleaning solutions. The latest software update (release 2.19b) optimises the use of these cleaning solutions, particularly for managing blanking issues associated with latex reagents. Simultaneous readings at nine wavelengths ensure analytical versatility, allowing for accurate and reliable measurements for a variety of assays.

The user-friendly interface of K450TX makes it accessible and easy to operate, even for less experienced users. Its intuitive software and ergonomic design ensure smooth operation, enabling laboratories to achieve consistent, high-quality results while maintaining efficiency and reliability.

The Menarini/ARKRAY ADAMS A1c HA-8180T (HA-8180T) (ARKRAY, Inc., Kyoto, Japan) is an automated benchtop analyser specifically designed for the measurement of HbA1c and the detection of haemoglobin variants, including S, C, and F. The instrument features a sample capacity of up to 100 per run, accommodating primary sample tubes with cap-piercing functionality. To ensure accuracy, the HA-8180T incorporates a mechanism to spin the samples, preventing blood sedimentation before analysis. The results are calculated based on the ratio of the HbA/HbA total, calibrated and reported in both International Federation of Clinical Chemistry (IFCC) units (whole numbers) and National Glycohemoglobin Standardization Program (NGSP) units (one decimal place). In addition to HbA1c, the analyser identifies haemoglobin variants, such as S, C, and F, reporting their respective percentages alongside HbA1c. While variants D and E are detected, their HbA1c values and percentages are not reported.

### 2.3. Reagents and Control Materials

The reagents used on K450TX were from Biolabo SAS (Maizy, France), specifically the HbA1c Turbidimetric Immunoassay (Ref. 22010/22011), HbA1c Standard Set (Ref. 22012), and HbA1c Control Set (Ref. 22013).

For the HA-8180T, the reagents comprised two calibrators, one with low concentration and one with high concentration, alongside the ARKRAY analytical column. Additionally, the analysis required three specific buffers (80A, 80B, and 80CV), which are integral to the chromatographic separation and measurement processes inherent to the HPLC technology.

### 2.4. Patient Samples

Blood samples were randomly selected from routine clinical laboratory accesses at the tertiary care Regional Hospital “San Salvatore” of L’Aquila. Venous blood was drawn under standardised conditions using a 20 G straight needle and collected in 3 mL siliconised vacuum tubes with minimal stasis (1–5 min). Following a routine laboratory protocol, the whole venous blood was haemolysed according to the manufacturer’s instructions and then subjected to analytical procedures. All specimens were transferred from labelled collection tubes to anonymised tubes, aliquoted, stored at temperatures between +2 °C and +8 °C until analysis to preserve the samples’ integrity, and analysed within 24 h. The analysers and reagents used for this study were employed according to the manufacturers’ instructions.

This study relied on anonymised leftover serum samples that were originally scheduled for disposal after routine clinical analysis, adhering to the principles outlined in the Helsinki Declaration and FDA guidelines for in vitro diagnostic device studies using leftover specimens, as they were originally intended for disposal after clinical testing.

### 2.5. Method Comparison

Comparability testing was conducted in accordance with the CLSI EP09C guideline “Measurement procedure comparison and bias estimation using patient samples” [36,38]. A total of 178 samples for HbA1c, spanning a broad analytical range, were analysed simultaneously on the Biolabo Kenza 450TX and the Menarini HA-8180V systems. The consistency of the data obtained from the two analysers was assessed using several statistical approaches.

The Passing–Bablok regression method was used to assess the degree of agreement between the methods. To confirm the validity of the Passing–Bablok linear regression, the Cusum test for linearity was employed [36,39,40]. Outlier detection was performed using Tukey’s test, Grubbs double-sided test, and generalised ESD test, while the data’s normality was ascertained with the Shapiro–Wilk test. However, the analysis did not reveal the presence of any outliers. The Bland–Altman difference plot was used to calculate the mean bias and limits of agreements (2.5 and 97.5 percentiles). The bias was calculated with 95% confidence intervals (CIs), and predefined total allowable error (ATE) limits were applied to assess acceptability. The ATE limits were set at ±5 mmol/mol (±0.46%), which is the minimum difference that would lead to a change in therapy. The lower and upper limits (2.5 and 97.5 percentiles) were calculated following the CLSI EP21 guideline and compared to the ATE limits. Additionally, a mountain plot was generated to provide a detailed visualisation of the distribution of the differences between the two methods in percentiles.

The concordance correlation coefficient (*ρ_c_*), as outlined by Lin [41], was calculated to evaluate the degree of concordance between the paired observations along the identity line. The *ρ_c_* combines precision (*ρ*), represented by Pearson’s correlation coefficient, and accuracy (*C_b_*), which quantifies the deviation of the fitted regression line from the theoretical identity line. The strength of agreement based on *ρ_c_* is categorised as follows: <0.90, poor; 0.90–0.95, moderate; 0.95–0.99, substantial; and >0.99, nearly perfect.

Scatter plots of the data were generated using a heat plot to provide a visual representation of the concordance between the two systems in relation to the theoretical line of identity. This method also emphasises the density and distribution of sample measurements across the analytical range, thereby enabling an intuitive assessment of agreement. Data points that closely align with the identity line indicate strong concordance, while deviations or clustering may suggest potential discrepancies.

The bias at predefined medical decision levels was estimated using the standard bootstrap confidence interval analysis, imposing 1000 bootstrapping replications in accordance with CLSI guideline EP09C [36].

### 2.6. Imprecision Evaluation

The imprecision was evaluated in accordance with the CLSI EP05-A3 guideline [35], using a 5 × 5 experimental design. Three concentration levels, low (35 mmol/mol, 5.4%), medium (76 mmol/mol, 9.1%), and high (112 mmol/mol, 12.4%), of the HbA1c normal and pathological lyophilised control samples were tested with both NGSP (%) and IFCC (mmol/mol) units. For each level, five replicates were measured within a single run over five consecutive days. All samples were processed within 4 h of reconstitution to ensure stability. The coefficients of variation (*CV*%) were calculated for repeatability (*CV_r_*%), between-day precision (*CV*_day_%), and within-laboratory precision (*CV_wl_*%).

### 2.7. Calculating the Probability of Risk of Misinterpretation

Based on the intralaboratory imprecision (*CV_wl_*), the estimated bias towards clinical decision values derived from the bootstrapping of patient clinical samples and the biological variabilities within and between subjects, it was possible to estimate the probability of risk associated with the clinical misinterpretation of analytical results. The calculation relied on the normalisation of the distribution of measurement uncertainties and biological variations, followed by the determination of the Z-values. These Z-values were subsequently converted to probability percentages of interpretative error using the cumulative table of the standard normal distribution [42]. This method was employed to calculate the probability of the risk of misinterpreting the results for HbA1c when utilised for monitoring and diagnosis with the equations presented in Table 1. The calculations were conducted for the risk of over/underdiagnosis in diagnosis and over/undertreatment in monitoring.

Where Δ is the minimum difference between two consecutive HbA1c measurements leading to a change in therapy and is equivalent to 5 mmol/mol (0.46%); *U* is the upper limit of HbA1c that defines diabetic disease and is equivalent to 47 mmol/mol (6.4%); and *L* is the lower limit of HbA1c that excludes diabetic disease and is equivalent to 39 mmol/mol (5.7%). *BV_w_* is the within-subject biological variability in HbA1c (1.6% for IFCC and 1.2% for NGSP), while *BV_b_* is the between-subject biological variability in HbA1c (7.1% for IFCC and 5.4% for NGSP) [43]. B is the bias associated with the measurement of HbA1c, and *CV_wl_* is the intralaboratory imprecision. *R* is equal to any true value of HbA1c.

To test the impact of analytical variability and biological variations on the probability of misinterpreting the HbA1c result, the same calculations were performed on an “ideal test”, where the measurement uncertainty bias and imprecision were settled to zero.

### 2.8. Software

The statistical analysis was performed using MedCalc version 18.2.1 (MedCalc Software, Ostend, Belgium) and OriginPro version 8.5.1 (OriginLab Corporation, Northampton, MA, USA).

### 2.9. Operating Temperature

All tests were performed by constantly monitoring the ambient temperature between 23 °C and 27 °C.

## 3. Results

### 3.1. Comparability Testing

**Figure 1:**
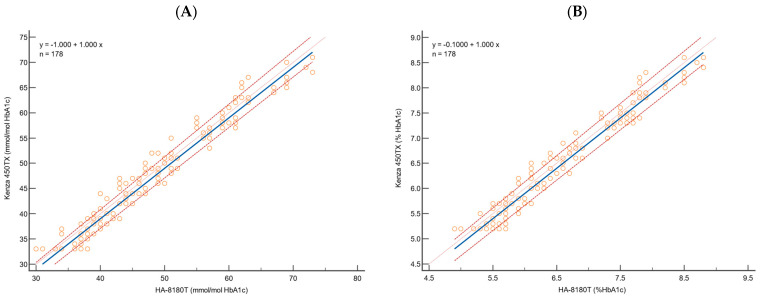
Passing–Bablok regression analysis for HbA1c, for 178 samples, using (**A**) IFCC and (**B**) NGSP to evaluate the correlation between HA-8180T (*x*-axis) and Kenza 450TX (*y*-axis). The blue solid line represents the regression line, the orange dotted line is the theoretical identity line, and the red dashed lines are the 95% confidence interval.

**Figure 2:**
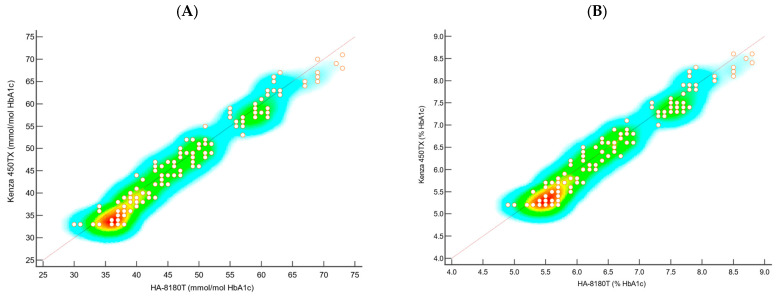
Scatter plots of the data using (**A**) IFCC and (**B**) NGSP illustrate the concordance between the two systems relative to the identity line, highlighting the data density and distribution. Points near the identity line indicate strong agreement, while deviations or clustering suggest potential discrepancies.

**Figure 3:**
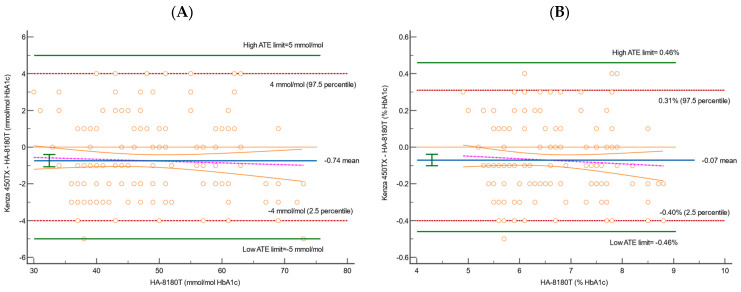
Bland–Altman difference plots for HbA1c for 178 samples, using (**A**) IFCC and (**B**) NGSP to evaluate the correlation between HA-8180T and Kenza 450TX. Blue solid lines represent the mean percentage difference; green horizontal lines are the ATE limits; orange dotted lines are the theoretical identity line; red dashed lines are the 95% confidence interval; and pink dashed lines are the regression line of the differences.

The analytical performance of the Biolabo Kenza 450TX immunoturbidimetric method was compared with the reference Hb-HPLC system (Menarini ADAMS A1c HA-8180T) for HbA1c determination. A total of 178 clinical samples from patients, covering a broader range of HbA1c concentrations, were analysed using multiple approaches, including Passing–Bablok regression (Figure 1 and Table 2), scatter plot (Figure 2), Bland–Altman difference plot (Figure 3), and the correlation coefficient of concordance (Table 3).

A bootstrap analysis (Table 4) was performed to assess the agreement between the two methods at the medical decision limits.

The bootstrap analysis confirmed a consistent and clinically acceptable bias across all tested HbA1c decision limits, for both the NGSP (%) and IFCC (mmol/mol) values. The modelled results obtained using the Kenza 450TX displayed a constant absolute bias of −0.10% (NGSP) and −1.00 mmol/mol (IFCC) at each medical decision point, including 5.7%, 6.4%, 7.0%, and 8.0% NGSP, and their IFCC equivalents, with narrow 95% confidence intervals. These results indicate excellent agreement with the reference method across clinically relevant thresholds, confirming both the analytical robustness and the clinical applicability of the immunoturbidimetric method throughout the decision-making range. Moreover, no proportional bias was observed, as the difference between the two methods remained constant across the tested concentration range.

The Passing–Bablok regression analysis demonstrated a strong correlation between the immunoturbidimetric method and the HPLC reference system for both NGSP (%) and IFCC (mmol/mol) values (Table 2, Figure 1). For instance, the slope was 1.00 (95% CI: 1.00 to 1.04) for both, indicating no proportional deviation from the theoretical line of identity. A slight systematic constant deviation was observed. For HbA1c expressed in NGSP (%), the intercept was −0.10 (95% CI: −0.33 to −0.1), while for IFCC (mmol/mol), the intercept was −1.00 (95% CI: −2.91 to −1.00). The minor constant deviation was confirmed by the 95% confidence interval (CI), which does not contain the theoretical 0 value.

The Cusum test for linearity confirmed no significant deviation from linearity with *p*-values above the significance threshold (NGSP: 0.27; IFCC: 0.08), confirming the linearity assumption of the Passing–Bablok regression model. The Spearman’s rank correlation coefficient showed a strong correlation (NGSP: 0.976; IFCC: 0.979) with a narrow 95% CI interval.

The scatter plots in Figure 2A,B, representing heat plots, were used to compare the HbA1c values obtained from the Kenza 450TX and the HA-8180T systems. These plots visualise the density and distribution of sample measurements across the entire analytical range, providing a visual assessment of the agreement between the two methods. The heat plot for the NGSP (%) values (Figure 2A) shows a high density of data points aligning closely along the identity line, indicating strong agreement between the two methods. No significant clustering or deviations from the identity line were observed, suggesting minimal bias and excellent concordance across the tested range. Similarly, the heat plot for the IFCC (mmol/mol) values (Figure 2B) confirms a consistent distribution of data points along the identity line, with no apparent outliers or systematic deviations. The uniformity of the density further supports the comparability of the Biolabo Kenza 450TX with the reference method, even at higher concentration levels.

The concordance correlation coefficient (*ρ_c_*) supported the Passing–Bablok findings, indicating substantial agreement between the two systems. The results are shown in Table 3. For NGSP (%), the *ρ_c_* value was 0.9741 (95% CI: 0.9653 to 0.9806), while for IFCC (mmol/mol), it was 0.9753 (95% CI: 0.9670 to 0.9816).

The Bland–Altman analysis, shown in Figure 3 and Table 5, provided further insight into the differences between the two systems.

The mean bias and the 95% CI were calculated for both the NGSP (%) and IFCC (mmol/mol) values, along with their corresponding limits of agreement (2.5th and 97.5th percentiles). The bias for NGSP was −0.07% (95% CI: −0.10 to −0.04), indicating a slight underestimation by the Kenza 450TX compared to the reference method. The lower limit of agreement (2.5th percentile) was −0.40% (95% CI: −0.46 to −0.40), and the upper limit (97.5th percentile) was 0.31% (95% CI: 0.30 to 0.40). These values fall within the predefined total allowable error (ATE) limits of ±0.46%, confirming that the Kenza 450TX meets the acceptability criteria. Similarly, for IFCC, the bias was −0.74 mmol/mol (95% CI: −1.01 to −0.40), showing a slight underestimation compared to the HPLC method. The lower limit of agreement was −4.00 mmol/mol (95% CI: −5.00 to −4.00), and the upper limit was 4.00 mmol/mol (95% CI: 3.58 to 4.00). These values also fall well within the allowable error limits of ±5 mmol/mol, demonstrating that the Kenza 450TX provides clinically acceptable results. At the predefined medical decision limits, the differences fell well within the acceptable ATE limits, with no significant deviations observed across the range of measurements.

The bootstrap analysis was used to evaluate the bias at specific decision levels. The estimated bias at each medical decision limit was −1.0 mmol/mol and −0.1%. All values reside within clinically acceptable limits (Table 4), thereby affirming the consistency of the Kenza 450TX across critical clinical thresholds.

**Figure 4:**
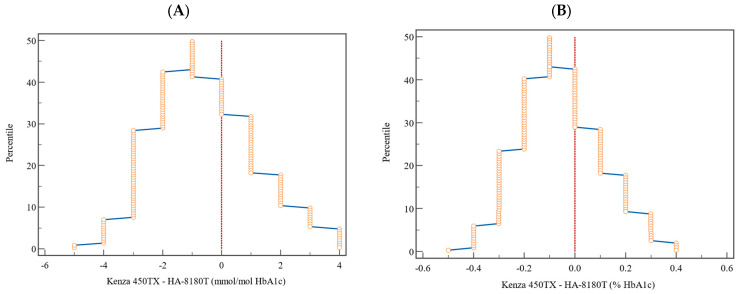
Graphical representation by mountain plot of the differences and relative percentile for the HbA1c values expressed (**A**) in percentage (NGSP) and (**B**) in mmol/mol (IFCC). The vertical dotted lines represent the reference to a zero-difference value between the two methods.

The mountain plot (Figure 4) provides a detailed view of the distribution of the differences in terms of percentiles. For HbA1c, expressed in both NGSP (%) and IFCC (mmol/mol) units, the differences were symmetrically distributed around the zero-difference line, indicating minimal deviations between K450TX and HA-8180T. The plot confirmed the absence of significant discrepancies across the analytical range, further validating the clinical comparability of the two methods.

### 3.2. Imprecision Studies

The imprecision was assessed following the CLSI EP05-A3 protocol, with the percentage of coefficient variation (*CV*) calculated for repeatability (*CV_r_*), between-day precision (*CV_day_*), and within-laboratory precision (*CV_wl_*). The results, summarised in Table 6, demonstrate excellent precision across all tested levels for both NGSP (%) and IFCC (mmol/mol) units. Specifically, for NGSP (%), the *CV_wl_* ranged from 1.26% at the low level (5.4%) to 2.00% at the high level (12.4%). Similarly, for IFCC (mmol/mol), the *CV_wl_* ranged from 2.1% at the low level (35 mmol/mol) to 2.4% at the high level (112 mmol/mol). These values were consistently clinically acceptable thresholds, demonstrating the reliability of the Biolabo Kenza 450TX system for HbA1c determination.

### 3.3. Risk of Misinterpretation

**Figure 5:**
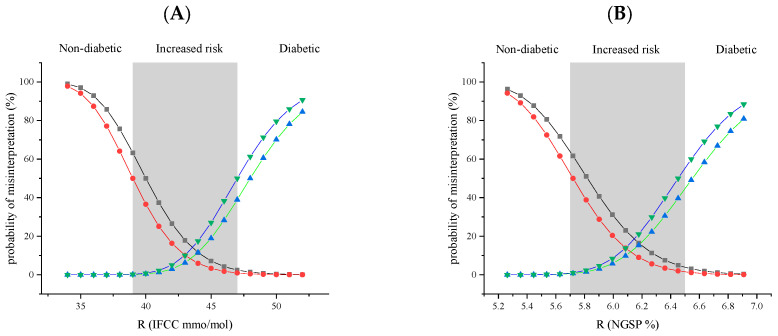
Graphical representation of the cumulative impact of the analytical uncertainty and biological variation on the probability of misinterpreting the HbA1c calculated for the immunoturbidimetric assay with K450TX versus an “ideal test”, where the analytical uncertainty, bias, and imprecision are set to zero. Black squares, K450TX overestimation; red circles, “ideal test” overestimation; blue triangles facing up, K450TX underestimation; and green triangles facing down, “ideal test” underestimation. The grey vertical band identifies the area between the medical decision limits for increased risk of diabetes: (**A**) IFCC 39–47 mmol/mol; (**B**) NGSP 5.7–6.5%. The estimated values are reported in Table S1 (Supplementary Data).

The probability of misinterpretation risk of the HbA1c results was evaluated using the methodology outlined above, which normalises measurement uncertainty distributions, calculates Z-values, and converts these to probability percentages using the standard normal cumulative distribution. To assess the impact of the analytical variability on the interpretative risk when the HbA1c results are employed for diagnosis or monitoring, we conducted calculations for the immunoturbidimetric method under evaluation (K450TX) compared with an idealised test, where the measurement uncertainty (bias and imprecision) was set to zero. The results demonstrate a high degree of consistency between the probability risk values for overestimation and underestimation for the K450TX and the ideal test when applied for diagnosis (Figure 5 and Table S1).

For example, at a true (*R*) value of HbA1c at the medical decision point of 47 mmol/mol, the risk of obtaining an overestimated HbA1c value below 39 mmol/mol is a 2.5% probability for K450TX and 1.0% for an ideal test without analytical uncertainty. Similarly, at a true value of 39 mmol/mol, the probability of obtaining an underestimated value that exceeds the medical decision point of 47 is 0.1% for K450TX and 0.2% for an ideal test. As can easily be observed in Figure 5, the cumulative probability of misinterpretation for K450TX and an ideal test is approximately the same. Across all *R* values analysed, the discrepancies between the K450TX immunoturbidimetric method and the ideal test are minimal, with no clinically significant differences.

The probability of misinterpretation was also calculated when using the HbA1c results to monitor the disease or to assess potential therapeutic changes (Table S2). In this case, the probability of misinterpreting the result for the K450TX immunoturbidimetric method is zero for every possible value of *R*. Thus, a comparison with an ideal test was unnecessary.

## 4. Discussion

The glycated haemoglobin (HbA1c) test is a pivotal biomarker in diabetes care. It is widely used to assess the risk of diabetes and monitor treatment efficacy. Over the past two decades, advancements in HbA1c testing have significantly improved diabetes management. However, the lack of standardisation across the numerous available methodologies, each with distinct reference ranges and reporting protocols, continues to pose challenges in ensuring comparability and reliability. This variability can impact the clinical decision making, establishment of universal benchmarks, and interpretation of research findings.

High-performance liquid chromatography is considered the reference standard for HbA1c measurement because of its accuracy and reliability. However, its high operational costs, complexity, and dependence on skilled personnel and specialised equipment limit accessibility, particularly in low-resource settings. These limitations highlight the urgent need for alternative methods that are affordable, cost-effective, user-friendly, and capable of providing results that closely correlate with HPLC.

Among the emerging alternatives, the immunoturbidimetric technique has attracted attention for its simplicity, efficiency, and affordability. It has proven particularly useful in resource-constrained environments, providing rapid and reliable results suitable for widespread use. Furthermore, it can be implemented and/or adapted to other clinical chemistry analysers, enhancing its applicability and integration into existing laboratory workflows. Therefore, it serves as a practical solution for expanding access to HbA1c testing.

The present study evaluates the analytical and clinical performance of the Biolabo Kenza 450TX, an immunoturbidimetric method for HbA1c determination, to assess its viability as a practical and efficient alternative to HPLC systems. Addressing the critical need for affordable yet accurate HbA1c testing methods, this analysis aims to contribute to the ongoing efforts to improve global standardisation and accessibility in diabetes care.

This study’s findings demonstrate that the Biolabo Kenza 450TX immunoturbidimetric method is a reliable alternative to the HPLC-based Menarini ADAMS A1c HA-8180T system for determining HbA1c. It also demonstrated strong analytical comparability across multiple evaluation criteria.

Passing–Bablok regression analysis emphasises the strong agreement of the immunoturbidimetric method with the reference system across a broad range of HbA1c concentrations. It demonstrated a robust correlation, with slopes of 1.00 for both HbA1c NGSP (%) and HbA1c IFCC (mmol/mol), and minor constant deviations, indicating negligible systematic bias. Furthermore, the results show a nearly perfect correlation between the two methods and are consistent with previous studies that have reported strong correlations between immunoturbidimetric methods and HPLC for HbA1c measurement [33]. In addition, our results are perfectly consistent with the data provided by the manufacturer, who report a slope of 1.0 and an intercept of 0.0 for HbA1c NGSP (%). The minor discrepancy observed in the intercept is negligible, especially considering the increased robustness of our analysis, which was performed on a larger dataset of 178 samples compared to the 40 samples analysed by the manufacturer.

The findings are corroborated by the concordance correlation coefficients (*ρ_c_*), which are recorded at 0.9741 for HbA1c NGSP (%) and 0.9753 for HbA1c IFCC (mmol/mol). These values further underscore the significant agreement and consistency between the two analytical methods. Such coefficients are comparable to those documented in analogous studies that evaluate the concordance between immunoturbidimetric assays and high-performance liquid chromatography (HPLC) [20].

The bootstrapping analysis conducted to estimate the bias at specific decision levels revealed minimal differences between the two methods (−1.00 mmol/mol and −0.10%). For example, at an HbA1c NGSP level of 6.4%, the Kenza 450TX exhibited a bias of −0.10%, corresponding to a relative difference of −1.56%. This minimal bias is within clinically acceptable limits and aligns with findings from other comparative studies [1]. The analysis reinforces the reliability of the immunoturbidimetric method. This consistency underscores the suitability of the Kenza 450TX for HbA1c measurement in routine clinical practice.

The Bland–Altman analysis indicated that the mean bias recorded (−0.07% for NGSP and −0.74 mmol/mol for IFCC) remains within acceptable parameters, and the overall variability identified is well within the predefined total allowable error (ATE) (Figure 3 and Table 5). The mountain plots, illustrated in Figure 4, demonstrate a symmetric distribution of differences, further substantiating these findings and corroborating the accuracy and reliability of the Kenza 450TX system for consistent HbA1c measurement in routine laboratory environments, particularly at critical clinical decision thresholds.

The Bland–Altman analysis revealed minimal and clinically insignificant biases between the two systems, with the agreement limits remaining within acceptable ranges for both the NGSP and IFCC values. This reinforces the reliability and comparability of the Biolabo Kenza 450TX for routine HbA1c assessment. Furthermore, the differences were uniformly distributed across the measurement range, exhibiting narrow 95% confidence intervals that are consistent with prior research. The regression line through the differences between the two methods corroborated the findings observed in the Passing–Bablok analysis, confirming the absence of proportional systematic deviation. These results further underscore the comparability of the two systems, implying that the immunoturbidimetric and HPLC methods are clinically interchangeable, as supported by analogous analytical studies documented in the literature [1].

The scatter plots depicted as heat plots (Figure 2A,B) reveal significant insights into the consistency of the Kenza 450TX across the analytical range. Both the NGSP (%) and IFCC (mmol/mol) values exhibited data points closely adhering to the line of identity, showing no notable clustering or deviations. This indicates minimal bias and strong concordance. These visual representations reinforce the statistical findings, demonstrating that the immunoturbidimetric method provides reliable and precise HbA1c measurements that closely align with those from the reference HPLC system.

Imprecision studies have revealed that the Kenza 450TX exhibited coefficient of variation (*CV*) values below 2.5% across low, medium, and high HbA1c levels, thereby demonstrating excellent repeatability and intermediate precision. It is recommended that the precision of HbA1c assays be maintained at less than 2.5% *CV*, as specified by the IFCC working group for HbA1c standardisation [20,44]. The robust analytical performance observed across all tested levels substantiates the use of the immunoturbidimetric method as a viable and cost-effective alternative to HPLC for HbA1c determination, particularly within the context of routine clinical practice. Moreover, the results obtained from this study in terms of imprecision are in line with the manufacturer’s specifications. Specifically, when comparing the within-run precision results near medical decision levels, our findings show lower values than those reported by the manufacturer, who indicate values of 1.3% for the low level, 1.0% for the normal level, and 1.5% for the high level.

The incorporation of a mean coefficient of variation of 2.2%, along with bias values derived from a rigorous statistical methodology applied to a substantial and diverse dataset, substantiates the analytical reliability of the Biolabo Kenza 450TX. This method’s performance not only satisfies the stringent requirements of laboratory environments but also adapts proficiently to the practical demands of clinical settings, thereby affirming its appropriateness for regular use in determining HbA1c levels.

The assessment of the risk probability associated with misinterpreting HbA1c results supports the established understanding that this analytical result is reliable when used to monitor disease progression. However, there are significant challenges in applying it to the diagnosis of diabetes mellitus.

A comparative analysis of the probability of risk linked to misinterpretation in relation to an ideal test occurring in the absence of measurement uncertainty substantiates the aforementioned difficulties. As illustrated in Figure 5 and Table S1, the discrepancies between the immunoturbidimetric method being evaluated and the ideal test are deemed insignificant for each true value, *R*, of HbA1c.

The differences between K450TX and the ideal test fall within a range unlikely to significantly impact clinical interpretation. For example, in the area near diagnostic thresholds (39–47 mmol/mol), where clinical decisions are most critical, the variation in probabilities of overestimation and underestimation between the two methods remains minimal. Despite the presence of analytical imprecision (*CV_wl_*) and bias (*B*) with the K450TX, this method demonstrates a performance comparable to the ideal test, suggesting that the analytical characteristics of K450TX do not significantly contribute to the risk of clinically misinterpreting HbA1c results. The predominant factor in consideration is the significant contribution of between-subject biological variability (*BV_b_*), particularly the relative discrepancy between its estimated value in a healthy population, 7.1%, and the within-subject biological variability (*BV_w_*), which is estimated at 1.6%. In light of this considerable divergence, all efforts to mitigate analytical uncertainty are deemed ineffective.

The individuality index, conceptualised by Eugene Harris five decades ago [45], indicates that the net ratio between *BV_w_* and *BV_b_*, excluding analytical uncertainty, yields an individuality index of 0.23. According to Harris’s model, this should be interpreted as a highly individual marker (value ≤ 0.6), leading to the conclusion that comparing it with reference values established for a healthy population is not appropriate. Instead, the result of testing the same marker is more valuable for monitoring the disease in the same individual, considering only analytical uncertainty and within-subject biological variability. The final case is confirmed by calculating the probability associated with the risk of misinterpretation during the monitoring process. As illustrated in Table S2, the probability of misinterpretation for the immunoturbidimetric method under examination is zero for any value of HbA1c (*R*).

Although high-performance liquid chromatography (HPLC) remains the gold standard for measuring HbA1c because of its specificity, the Kenza 450TX offers a practical and cost-effective alternative, particularly beneficial for laboratories with limited resources. In particular, the Kenza 450TX analyser combines high analytical throughput (up to 420 tests per hour) with minimal maintenance needs and ease of use. Its ability to measure HbA1c alongside routine biochemistry on a single platform reduces both the turnaround time and workflow complexity. These features are particularly valuable in the context of serial HbA1c monitoring, where frequent and timely assessments are essential for adjusting treatment and supporting long-term glycaemic control. However, the study’s single-centre design and the exclusion of samples exhibiting rare haemoglobinopathies limit its generalisability. Future research efforts should aim to expand validation across diverse populations and investigate performance in the presence of haemoglobin variants. However, the manufacturer reports that common variants, such as HbA2, HbC, and HbS, do not interfere with the assay within validated concentration ranges. Moreover, the absence of an experimental evaluation of the potential analytical interferences, such as icterus, lipaemia, or haemoglobin carbamylation, represents a limitation of the present study and should be addressed in future investigations. The Biolabo Kenza 450TX provides reliable and clinically acceptable HbA1c measurements, establishing it as a valuable tool for diabetes management across various healthcare settings.

## 5. Conclusions

The evaluation of analytical performance represents a fundamental aspect of the quality control process within clinical laboratories, guaranteeing not only the reliability of the results but also the overarching optimisation of efficiency and cost-effectiveness. In this regard, the present study is dedicated to the assessment of the Biolabo Kenza 450TX immunoturbidimetric system for the determination of HbA1c, highlighting its capacity to enhance laboratory operations and meet clinical diagnostic requirements.

The analysis assessed the essential parameters of the Kenza 450TX, encompassing its precision, accuracy, concordance with the HPLC reference system, and its appropriateness for routine clinical application. The results indicate that the immunoturbidimetric method yielded outcomes that were comparable to those obtained from the HPLC reference method, exhibiting exceptional precision and clinically acceptable levels of bias.

One primary feature of the system is its capacity to integrate analytical efficiency with operational effectiveness. By enabling HbA1c determination within a multifunctional biochemistry analyser, the Kenza 450TX eliminates the necessity for separate instruments, thereby reducing operational costs, optimising laboratory space, and simplifying equipment management. In addition, its low reagent consumption, minimal maintenance needs, and compatibility with routine biochemistry workflows contribute to significantly reduced operational costs compared to dedicated HbA1c platforms such as HPLC systems. These benefits are particularly significant in resource-limited settings, such as decentralised laboratories or facilities in low-income areas, where economic sustainability and accessibility are crucial. Furthermore, the reliability of the K450TX was demonstrated through various evaluations, including regression analysis, bias measurements, and precision studies. Its performance consistently met the requirements for diabetes management, proving it to be a dependable alternative to HPLC systems.

In our professional assessment, the calculations regarding the probability of misinterpretation were predicated upon the standardisation of the measurement uncertainty distributions, thereby ensuring robust comparability among the various methodologies. The evaluation indicates that the K450TX method operates in a manner nearly indistinguishable from that of an ideal test, exhibiting zero bias and imprecision. The negligible differences noted are not clinically significant, thereby reinforcing the robustness of K450TX and fostering confidence in its analytical performance.

In conclusion, the Biolabo Kenza 450TX emerges as a cost-effective, reliable, and efficient solution for the determination of HbA1c levels, rendering it exceptionally suitable for laboratories operating with constrained resources. Its seamless integration into pre-existing workflows and its robust analytical performance substantiate its viability as a practical instrument for augmenting access to diabetes monitoring and enhancing patient care on a global scale.

## Figures and Tables

**Table 1:** The equations used to calculate the probability of clinical misinterpretation of HbA1c for the diagnosis and monitoring of diabetic disease.

HbA1c Application	Clinical Interpretation Error Category	Equations for Calculating Z-Values
Diagnosis	Underdiagnosis	$z=\frac{\left(R+B\right)-L}{\frac{R}{100}\sqrt{{CV}_{wl}^{2}+{BV}_{w}^{2}+{BV}_{b}^{2}}}$
	Overdiagnosis	$z=\frac{U-\left(R+B\right)}{\frac{R}{100}\sqrt{{CV}_{wl}^{2}+{BV}_{w}^{2}+{BV}_{b}^{2}}}$
Monitoring	Undertreatment	$z=\frac{∆-B}{\frac{R}{100}\sqrt{2{CV}_{wl}^{2}+{BV}_{w}^{2}}}$
	Overtreatment	$z=\frac{∆+B}{\frac{R}{100}\sqrt{2{CV}_{wl}^{2}+{BV}_{w}^{2}}}$

**Table 2:** Comparison of the HbA1c determination methods between Menarini ADAMS A1c HA-8180T and Biolabo Kenza 450TX by Passing–Bablok analysis performed on 178 human clinical patient samples.

Parameter	HA-8180T *^a^* (Range)	K450TX *^a^* (Range)	Slope (95% CI)	Intercept (95% CI)	Correlation Coefficient *^b^* (95% CI)	CUSUM Test *^c^*
HbA1c NGSP (%)	6.4 (4.9 to 8.8)	6.4 (5.2 to 8.6)	1.00 (1.00 to 1.04)	−0.10 (−0.33 to −0.1)	0.976 (0.967 to 0.982)	0.27
HbA1c IFCC (mmol/mol)	47 (30.0 to 73.0)	46.5 (33.0 to 71.0)	1.00 (1.00 to 1.04)	−1.00 (−2.91 to −1.00)	0.979 (0.971 to 0.984)	0.08

*^a^* Median value. *^b^* Spearman’s rank correlation coefficient. *^c^* CUSUM test for linearity, which evaluates the fit of a linear model to the data.

**Table 3:** Comparison of the HbA1c determination methods between Menarini ADAMS A1c HA-8180T and Biolabo Kenza 450TX using the concordance correlation coefficient method.

Parameter	Pearson Correlation Coefficient (*ρ*)	Bias Correction Factor (*C*_b_)	Concordance Coefficient (*ρ*_c_) (95% CI)	Strength of Agreement ^a^
HbA1c NGSP (%)	0.9767	0.9973	0.9741 (0.9653 to 0.9806)	substantial
HbA1c IFCC (mmol/mol)	0.9777	0.9975	0.9753 0.9670 to 0.9816	substantial

^a^ The strength of the agreement can be extrapolated from the *ρ_c_* value, as follows: <0.90, poor; 0.90–0.95, moderate; 0.95–0.99, substantial; >0.99, almost perfect.

**Table 4:** Bias calculated at the specific decision levels obtained by standard bootstrap confidence interval approximation, calculated by imposing 1000 bootstrap replications (iterations: 1000; random number seed: 978).

Parameter	Decision Limit	Modelled Kenza 450TX (Bootstrap 95% CI)	Difference (Bootstrap 95% CI)	Relative Difference (Bootstrap 95% CI)
HbA1c NGSP (%)	5.7	5.60 (5.57 to 5.64)	−0.10 (−0.13 to −0.06)	−1.75% (−2.24 to −1.08%)
	6.4	6.30 (6.29 to 6.35)	−0.10 (−0.10 to −0.05)	−1.56% (−1.61 to −0.78%)
	7.0	6.90 (6.90 to 6.97)	−0.10 (−0.10 to −0.03)	−1.43% (−1.43 to −0.49%)
	8.0	7.90 (7.90 to 8.00)	−0.10 (−0.10 to −0.005)	−1.25% (−1.25 to 0.062%)
HbA1c IFCC (mmol/mol)	39.0	38.00 (37.45 to 38.10)	−1.00 (−1.55 to −0.90)	−2.56% (−3.97 to −2.31%)
	47.0	46.00 (45.50 to 46.45)	−1.00 (−1.50 to −0.55)	−2.13% (−3.19 to −1.17%)
	53.0	52.00 (51.59 to 52.74)	−1.00 (−1.41 to −0.25)	−1.89% (−2.67 to −0.48%)
	64.0	63.00 (62.96 to 64.27)	−1.00 (−1.03 to −0.27)	−1.56% (−1.62 to 0.43%)

**Table 5:** Bias was calculated on 178 human clinical patient samples by means of a Bland–Altman analysis performed by plotting on the *x*-axis the value measured with the reference method (Menarini ADAMS A1c HA-8180T) and on the *y*-axis the difference between the results obtained with the method to be validated (Biolabo Kenza 450TX) and the reference method. The lower 2.5 percentile and the upper 97.5 percentile were compared with the low and high ATE limits in accordance with CLSI EP21 “Evaluation of Total Analytical Error for Quantitative Medical Laboratory Measurement Procedures. 2nd Edition”.

	Bias (95% CI)	Lower P2.5 (95% CI)	Low ATE Limit (Interpretation)	Upper P97.5 (95% CI)	High ATE Limit (Interpretation)
HbA1c NGSP (%)	−0.07 (−0.10 to −0.04)	−0.40 (−0.46 to 0.40)	−0.46% (passed)	0.31 (0.30 to 0.40)	0.46% (passed)
HbA1c IFCC (mmol/mol)	−0.74 (−1.01 to −0.40)	−4.00 (−5.00 to −4.00)	−5 mmol/mol (passed)	4.00 (3.58 to 4.00)	5 mmol/mol (passed)

**Table 6:** The results of the imprecision study on Biolabo Kenza 450TX, with the percentage of coefficient variation (*CV*) calculated for repeatability (*CV_r_*), between-day precision (*CV_day_*), and within-laboratory precision (*CV_wl_*).

	Level	Mean	*CV_r_* (%)	*CV_day_* (%)	*CV_wl_* (%)
HbA1c NGSP (%)	Low	5.4	0.89	0.90	1.26
	Medium	9.1	0.68	1.46	1.61
	High	12.4	1.39	1.45	2.00
HbA1c IFCC (mmol/mol)	Low	35	1.5	1.5	2.1
	Medium	76	0.9	1.9	2.1
	High	112	1.7	1.8	2.4

## Data Availability

The original contributions presented in this study are included in the article/Supplementary Material. Further inquiries can be directed to the corresponding author.

## Supplementary Materials

The following supporting information can be downloaded at: https://www.mdpi.com/article/10.3390/diagnostics15080969/s1, Table S1: Calculation of the cumulative impact of analytical uncertainty and biological variation on the probability of misinterpreting HbA1c when used for diagnosis, calculated for the immunoturbidimetric assay with the K450TX versus an “ideal test”, where the analytical uncertainty, bias, and imprecision are set to zero. The grey horizontal bands identify the area between the medical decision limits for increased risk of diabetes: IFCC, 39–47 mmol/mol; NGSP, 5.7–6.5%; Table S2: Calculation of the cumulative impact of the analytical uncertainty and biological variation on the probability of misinterpreting HbA1c, when used for monitoring and calculated for the immunoturbidimetric assay with the K450TX. The grey horizontal bands identify the area between the medical decision limits for changing in therapy, as follows: IFCC, 53–64 mmol/mol; NGSP, 7.0–8.0%.
