# Supplementary material for: Evaluation of the Biolabo Turbidimetric Assay for Automated Determination of Haemoglobin A1c
Source: Diagnostics (Basel). 2025 Apr 10;15(8):969. doi: 10.3390/diagnostics15080969 (PMC12025445; doi:10.3390/diagnostics15080969)
Supplement: Supplementary file 1 [file diagnostics-15-00969-s001.zip › diagnostics-3542928-supplementary.pdf]

Table S1. Calculation of the cumulative impact of analytical uncertainty and biological variation on the probability of misinterpretation of HbA1c when used for diagnosis, calculated for the immunoturbidimetric assay on K450TX versus an "ideal test" where the analytical uncertainty, bias, and imprecision are set to zero. The grey horizontal bands identify the area between the medical decision limits for increased risk of diabetes: IFCC 39-47 mmol/mol and NGSP 5.7-6.5%.

			Probability risk (%)						
	overestimation		underestimation			overestimation		underestimation	
**R (IFCC)**	**K450TX**	**Ideal test**	**K450TX**	**Ideal test**	**R (NGSP)**	**K450TX**	**Ideal test**	**K450TX**	**Ideal test**
34	99.0	97.8	0.0	0.0	5.3	96.3	94.2	0.0	0.0
35	97.0	94.2	0.0	0.0	5.4	92.9	89.2	0.0	0.0
36	92.9	87.4	0.0	0.0	5.4	87.7	81.9	0.0	0.0
37	85.8	77.1	0.0	0.0	5.5	80.6	72.5	0.1	0.1
38	75.6	64.1	0.0	0.1	5.6	71.7	61.6	0.3	0.4
39	63.3	50.0	0.1	0.2	5.7	61.6	50.0	0.7	1.0
40	50.0	36.6	0.4	0.8	5.8	51.0	38.8	1.6	2.3
41	37.4	25.1	1.2	2.2	5.9	40.6	28.8	3.2	4.6
42	26.5	16.3	3.0	5.1	6.0	31.1	20.4	5.8	8.4
43	17.9	10.1	6.2	10.1	6.1	23.0	13.9	9.8	13.9
44	11.5	5.9	11.5	17.4	6.2	16.4	9.0	15.3	21.1
45	7.1	3.3	18.9	27.1	6.3	11.3	5.7	22.3	29.9
46	4.3	1.8	28.3	38.3	6.4	7.6	3.4	30.5	39.7
47	2.5	1.0	38.9	50.0	6.5	4.9	2.0	39.7	50.0
48	1.4	0.5	50.0	61.3	6.5	3.1	1.1	49.1	60.0
49	0.8	0.3	60.6	71.3	6.6	1.9	0.6	58.4	69.1
50	0.4	0.1	70.1	79.5	6.7	1.1	0.3	66.9	77.0
51	0.2	0.1	78.1	85.9	6.8	0.7	0.2	74.5	83.4
52	0.1	0.0	84.5	90.7	6.9	0.4	0.1	80.9	88.4

Table S2. Calculation of the cumulative impact of analytical uncertainty and biological variation on the probability of misinterpretation of HbA1c, when used for monitoring, calculated for the immunoturbidimetric assay on K450TX. The grey horizontal bands identify the area between the medical decision limits for changing in therapy: IFCC 53-64 mmol/mol and NGSP 7.0-8.0%.

	HbA1c IFCC (mmol/mol)			HbA1c NGSP (%)	
**R**	**overestimation**	**underestimation**	**R**	**overestimation**	**underestimation**
48	0.0	0.0	6.5	0.0	0.0
49	0.0	0.0	6.6	0.0	0.0
50	0.0	0.0	6.7	0.0	0.0
51	0.0	0.0	6.8	0.0	0.0
52	0.0	0.0	6.9	0.0	0.0
**53**	**0.0**	**0.0**	**7.0**	**0.0**	**0.0**
54	0.0	0.0	7.1	0.0	0.0
55	0.0	0.0	7.2	0.0	0.0
56	0.0	0.0	7.3	0.0	0.0
57	0.0	0.0	7.4	0.0	0.0
58	0.0	0.0	7.5	0.0	0.0
59	0.0	0.0	7.5	0.0	0.0
60	0.0	0.0	7.6	0.0	0.0
61	0.0	0.0	7.7	0.0	0.0
62	0.0	0.0	7.8	0.0	0.0
63	0.0	0.0	7.9	0.0	0.0
**64**	**0.0**	**0.0**	**8.0**	**0.0**	**0.0**
